# Reincluding: Providing Support to Reengage Youth who Truant in Secondary Schools

**DOI:** 10.5334/cie.47

**Published:** 2022-10-03

**Authors:** Delia Baskerville

**Affiliations:** 1 Victoria University of Wellington, NZ

**Keywords:** Truancy, reinclusion, teacher intentional behaviours, accommodating

## Abstract

Truancy, a complex, unresolved educational issue in countries with compulsory attendance policies, has the potential to cause further educational inequity in times of a global COVID-19 pandemic. At the time of this study, there was a paucity of research regarding youth perspectives of truancy compared to adult perspectives. To address this gap in truancy scholarship, data from a grounded theory study were used to indicate how a sample of 13 students who were truant in New Zealand explained their experiences of reinclusion in learning after persistent absences. Findings showed that support by a significant adult, not necessarily a teacher, and peers were vital in helping youth who are truant to develop a positive and determined attitude to reengage with learning. Results will support school leaders, counsellors, and teachers to further develop inclusive approaches that promote student well-being and achievement.

Truancy is a serious, longstanding social issue impacting negatively on student achievement and public safety. Youth who are truant (YwT) use the term “wagging” to explain truancy, which they defined as a common event happening in certain situations at specific times, involving both intentional disregard of school rules and absence from school (Baskerville, 2019). Bassey (2020) suggested that truancy refers to deliberate departure from school without parental consent. In other words, YwT are absent without justification (Keppens & Spruyt, 2020). Moreover, in countries where school attendance is compulsory, truancy is perceived as a delinquent act (Onyele, 2018). Currently “brazen” youth crime is causing concern around New Zealand (MSNZ, 2022).

The current global pandemic places increasing pressure on our education systems in general, including teachers and student attendance. Internationally, teachers already battle with the challenges of a broadening curriculum, increasing accountability, assessment and paperwork, reduced resourcing, and impaired health and well-being (Nilsson et al., 2015). Likewise, in New Zealand (NZ), issues in education relate to an aging teacher workforce, teachers’ unwillingness to take on leadership roles, retention of new teachers, teacher supply, and teacher specialist content knowledge (Education Government New Zealand, n.d.).

YwT are marginalized through experiences of diminished educational opportunities and a lifetime of associated problems. Thus, international research has found associations between truancy and underachievement (Khan et al., 2019), unemployment (Garry, 1996), delinquency, drug involvement, and criminal activity (Gerth, 2020; Javier et al., 2020), as well as school dropout and incarceration (Iverson et al., 2016).

Comparably, truancy in NZ is a serious problem, with truancy rates reaching their highest level during the last decade. For example, the 2019 attendance report (Ministry of Education [MoE], 2020a) shows that school attendance dropped substantially over the previous five years. In 2019, only 58% of students attended school regularly during Term 2 (i.e., 42% of students were absent from school for the equivalent of one or more days per fortnight). This was down from 69% in 2015. This large drop occurred across all regions, all ethnicities, all deciles (ratings used by the Ministry of Education to work out some funding for schools), and all age levels (MoE, 2020a).

The recent inquiry into school attendance presented to the House of Representatives in April of 2022 (School and Workforce Committee, 2022) proposed four recommendations: (a) the government prioritize the development and implementation of a school attendance strategy; (b) the strategy aim to improve school attendance; (c) the strategy target at least sustained regular attendance for 70% of students by 2024, and 75% by 2026; and (d) schools receive notification of the target and the intention to monitor the outcomes by Education Review Office (ERO). The Education Minister, Chris Hipkins, announced a pre-budget, 88m dollar million funding package to battle the truancy epidemic, particularly post-COVID (Newshub, 2022).

Initial analysis of NZ data collected during 2020 (MoE, 2020b) suggests that compared to 2019, senior secondary school students (ages 11–13) experienced significantly increased attendance due to COVID-19 post lockdown, perhaps due to students’ desires to experience normalcy, their motivation to catch up on National Certificate of Educational Achievement assessment opportunities lost during lockdown, and prospects to socialize with their peers (MoE, 2020b). However, many students whose attendance was most negatively impacted by COVID-19 did not necessarily have troubling attendance patterns at the beginning of the year. Existing inequities in school attendance seems to be substantially exasperated by COVID-19, particularly in Auckland (MoE, 2020b). Students are most likely to have reduced their attendance in response to COVID-19 if they are in earlier year levels (1 and 2), attend a low-decile school, are Pacific or Māori, or attend Māori-medium schools. Factors contributing to amplified barriers and stigma to attendance in students experiencing poverty may include (a) non-existent access to devices or connectivity, thereby excluding remote learning; (b) insecure housing situations; (c) greater dependence on perceived, less safe public transport; (d) insufficient funding for uniforms or learning materials; and (e) increased likelihood of health issues that create greater risk of being harmed by COVID-19 (MoE, 2020b). Because of this inequitable widening gap in opportunities to learn, the aim of this study was to raise awareness of the nature of support required for students to return to, and sustain, school attendance post truancy.

## Reincluding

*Reincluding* is an existing concept in the language of returning long-term absentee students to mainstream education. Thomas (2015, p. 191) suggested coining the term *reinclusion* for two reasons. First, reincluding reflects schools’ willingness to accommodate students, which he maintains is foundational to success. Second, reincluding suggests disadvantaged students no longer suffer exclusion from education. Thomas (2015) highlighted participants’ responses to inclusion from teachers and peers; that is, when reincluded in class, participants experienced contentment, visibility, academic success, and a sense of belonging to a place. Youth marginalized by truancy, excluded from classrooms, and subjected to changes in school policy without consultation, report that they require support to improve their attendance. To frame this discussion, three areas critical for supporting YwT – fostering collective responsibility, establishing inclusive environments, and providing support for marginalized youth to recommence attending class (Baskerville, 2019) – are presented one at a time.

## Fostering Collective Responsibility

The concept of “fostering collective responsibility” in relation to preventing truancy refers to the respectful positioning of students and the implementation of student-centered pedagogies to foster student engagement in learning, student autonomy, and strengthening of home-school relationships (Baskerville, 2019). Studies of youth with a history of truancy (Gase et al., 2016) suggest that schools have an opportunity to prevent truancy in four ways: (a) promoting school engagement and providing meaningful learning personalized to student interests and experiences; (b) responding to truancy more effectively (e.g., early interventions that establish clear boundaries and execute consequences); (c) addressing the root causes of truancy; and (d) consolidating staff and parent relationships. These practices improve parents’ capacity to understand school structures and use resources more effectively to support youth to reduce their truancy behaviour, suggesting that student-centered approaches help diminish truancy (Gase et al., 2016).

When YwT co-construct behaviour expectations with their teachers, they report a positive sense of self, positive relationships with teachers, and a sense of autonomy (McHugh et al., 2013). Such consultation, respect, and inclusion of students’ viewpoints contrasts with students who experience disrespectful social relationships, exclusion, and unsafe learning environments (Reid & McCallum, 2014; Strand, 2014; Syrjäläinen et al., 2015). Likewise, explicit teacher expectations also support youth functioning in classrooms. For example, specific teacher behaviours are essential to influence youth to accept responsibility for their behaviour and avoid blame for their exclusion (Lewis et al., 2012). Students excluded from class claim it is beneficial if teachers explain why exclusion is necessary, warn them before excluding them, and avoid just “telling them off.” They also recommend that teachers hold a follow-up conversation to highlight the impact of students’ misbehavior on classmates (Lewis et al., 2012). Excluded youth perceive that implementing such strategies would prevent them from setting out on a slippery slope of exclusion and underachievement and instead be successfully reintegrated into their classes and more likely accept that their misbehavior impacted their peers. They also felt that they would take more responsibility for their actions if teachers behaved reasonably to them (Lewis et al., 2012). Thus, teacher intentional professional behaviour appears to be a precursor for excluded youth accepting responsibility for their misbehaviors in addition to increasing the opportunities for their re-inclusion and achievement.

Furthermore, youth gain autonomy when they are consulted respectfully. For example, DaCosta (2006) advocated for a more viable student-centered approach to enhance student ownership, specifically student involvement in decision-making when co-designing school uniforms. She claimed that early involvement supports youth to learn to think about others empathetically, respond caringly to others’ thoughts and feelings, and find thoughtful and responsible solutions to problems. In other words, when teachers position youth respectfully to share power, youth experience autonomy (DaCosta, 2006). It appears then that the actions of teachers – consultation with students, inclusion of students in decision-making, blame avoidance, and explanations for discipline – contribute to collective responsibility and students gaining a sense of autonomy.

## Establishing Inclusive Learning Environments

The second critical area for supporting YwT, “establishing inclusive learning environments,” relates to the support provided to excluded students to help them develop a sense of belonging to school. It includes the experience of acceptance and connection to others, and the development of self-efficacy, autonomy, and feeling valued (Baskerville, 2019). Teachers who establish and monitor classroom environments through inclusive teaching and learning approaches provide contexts for meaningful learning. For example, Syrjäläinen et al. (2015) found that youth associated caring by their teachers with feelings of safety, joy, well-being, trust, and empathy. These youth wanted inclusive approaches such as acceptance of difference and building positive teacher-student relationships. Under such conditions, youth reported being able to build trust, receive support, and experience closeness with others. Inclusive teaching was also associated with the establishment of collective responsibility and the development of students’ sense of belonging (Syrjäläinen et al., 2015).

Similarly, alternative “enabling spaces” (O’Donovan et al., 2015, p. 645) – learning opportunities situated outside school classrooms in spaces within the school setting – supported previously excluded students to participate in practical, hands-on building projects. When students were supported to explore respectful relationships, to foster a sense of belonging, and to develop self-efficacy, time and space allocated for previously excluded students to connect with others contributed to meaningful learning occurring (O’Donovan et al., 2015). Likewise, the opportunity for students to engage in music making outside the classroom provided a meaningful school context with positive experiences (Stahl & Dale, 2013). Specifically, it supported youth to change their identities from failures to achievers because they were inspired to attend. Further, they persevered because they did not associate failure with loss of status, and the supportive music-learning environment helped them to recognize that their skill base was improving (Stahl & Dale, 2013). In addition, the context of an inspiring supportive, inclusive learning environment provided a place for them to attend. They experienced relationships that were positive and equal. Outside of class, the youth chose work that inspired them, their peers recognized them as successful, and their identities changed. In short, positive relationships, equal status, peer approval, and inspiring contexts for learning are the features of inclusive learning environments that support YwT to participate in learning and to experience success (Stahl & Dale, 2013).

Research related to establishing inclusive environments for YwT suggests that relevant, meaningful, purposefully designed curricula positions them favorably to achieve academically. YwT claimed that they needed to be safe, connect with others, and be seen as successful in order to belong at school and achieve (O’Donovan, et al., 2015; Stahl & Dale, 2013; Syrjalainen et al., 2015).

## Providing Support for Students to Recommence Attending Class

The third way to support youth – “providing support for students to recommence attending class” – refers to the nature of the social and emotional support that YwT need to encourage their attendance at school (Baskerville, 2019). Specific approaches provide support for youth to change. Youth recommend that teachers prevent student bullying, build positive teacher-student relationships, accept difference, and encourage closeness (Syrjäläinen et al. 2015). In this way, collective responsibility is emphasized, and every student receives the support necessary to feel a sense of belonging.

Delgado et al. (2016) reported that when students were nominated as a friend by peers and experienced the perception that they were amongst friends, their sense of belonging to school was enhanced, suggesting that safe learning environments and close reciprocal relations between students promote school belonging. However, Hallinan (2008) noted that, “it is teachers’ support more than youth friendships that increased youth liking of school” (p. 281). Hallinan concluded that when teachers provided social and emotional support to students through care, respect, and praise, they strongly influenced students’ attachment to school (Hallinan, 2008). This argument is supported by Edinburgh et al. (2013), who found that Hmong runaway girls were motivated to return home and try to change because of factors such as missing their younger siblings. However, these girls needed culturally relevant, youth-centered support to reunite with their families, implying that youth want support to reconcile differences with others. Such support involves the positioning of youth as culturally located beings, having friends, experiencing closeness to others, being accepted, respected, and praised, and feeling safe.

Across different countries then, youth perspectives on truancy provide insight into the impact of deficit adult thinking in terms of labelling, stereotyping, and imposed positioning. This imposed negative identity contributes to youth exclusion and marginalization. Youth perceive other school circumstances, such as unsafe learning environments, uninteresting curricula, lack of attention from teachers and others, and social rejection as contributing to diminished confidence and personal insecurity in classrooms. Youth understand they need support, care, reciprocity of relationships, peer approval, and equal status to promote school belonging and attendance.

However, at the time of this research, compared to other stakeholders (e.g., head teachers and parents), little was known about the nature of support required for YwT in NZ to return to class and reengage in learning. The intent of this study was to delve deeper into these circumstances in NZ and identify the nature of support required for YwT to reengage in learning so post-truancy we may begin to address the inequitable widening gap in opportunities to learn, exasperated by the current pandemic.

## Method

### Participants

This study about truancy, not school exclusion, draws on the data of a wider qualitative study. The aim of the present investigation was to determine how a sample of 13 youth experienced their world of returning to class after truanting and the social aspects of their circumstances; in other words, to understand their behaviours as they understood them. The paper concentrated on one subquestion of the wider study: how, and under what circumstances, are NZ youth able to reintegrate in class after persistent truanting?

The process of recruiting subjects was complex. That is, it took time to locate the 13 youth, 7 girls and 6 boys, aged between 13 and 18, because they were not attending school regularly. Participants were recruited using a poster that contained the researcher’s photograph, an invitation to participate, a description of the research, and the person to contact if they wanted to be interviewed. The posters were placed strategically in schools; for example, outside a counsellor’s office.

At the time of the study, seven participants lived in single-parent families, and two lived with their birth parents. Two girls lived separately from their siblings. Four participants (three girls and one boy) experienced mental illness; the three girls all exited school, one boy was on suspension, one girl left school at 16, and two girls continued to be truant. Six participants (four boys and two girls) returned to regular attendance. The inductive analysis of participant interviews intended the emergence of student voice.

### Design

Grounded theory (GT; Glaser & Strauss, 1967), the selected methodological framework, refers to “an educational process of events, activities, actions and interactions that occur over time” (Creswell, 2012, p. 423). Data gathered from individual semi-structured interviews, field notes, and memo writing provided evidence of conversations and observations of youths’ experiences of truancy in four secondary schools in NZ. Examples of themes covered in the interviews related to participants’ experiences of school, support received to attend class regularly, teachers’ attitudes to participants, and participants’ motivation to change.

Using GT to analyse the data, the goal was to discover a new theory that emerges from analysis of the data gathered from participants rather than interpreting the data in relation to prior conceptions that may be based in literature, or in the researcher’s preconceived beliefs. GT develops a rigorous theory by applying systematic procedures (Thistoll et al., 2016). The investigation, grounded in student data, presented an original process theory that identifies and explains a process of wagging (truancy) in four NZ secondary schools and clearly explained contextual boundaries. Three key components of GT (Glaser & Strauss, 1967) were implemented in conducting this study: (a) *constant comparison* of new data with earlier coded pieces of data to test and refine current interpretations and establish coherence between categories and their properties; (b) *theoretical sampling* served as a key strategy to purposefully select further participants and gain specific knowledge, thereby obtaining data clarify interpretations linked to gaps or ambiguities in the emerging theory; and (c) *theoretical saturation*, the point in the process of GT when the emerging theory reached stability and no further data were needed. The rigorous methodology of constant comparison and theoretical sensitivity ensured conceptual density, integration, and clarity (Glaser & Strauss, 1967).

### Procedure

Participants chose to take part in the study and selected a pseudonym to grant privacy to the words they spoke. Ethics approval gained from Victoria University Faculty of Education Wellington (Ethics Number RM19352) included permission to negotiate informed consent, voluntary participation, privacy, and confidentiality.

Participants were interviewed individually, given time to get to know the researcher, listen to the rationale for the research, and establish a rapport with the researcher to support them not to be nervous about sharing their experiences. Two interviews per student (for a total of 26) was planned, but only 20 interviews were completed, as some participants were unavailable for a second interview due to relocation, drug use, and disappearance. Through privileging student voice using GT methodology, rather than focusing on a single reality, I was able to compare similarities and differences in participants’ perspectives. This simultaneous gathering and analysis of participant interviews in the wider study produced a process theory of wagging that identified four stages of wagging: wagging-in-class, leaving, awakening, and reincluding (Baskerville, 2019). The process theory contributed to understanding the nature of wagging as a process and what YwT and adults needed to do to stop the process from happening, or to shortcut it. It also contributed knowledge about truancy in terms of the nature of wagging, causes of truancy, relationship pedagogy, repositioning of YwT, the experience of mattering, and the reinclusion of students into class after wagging. It is the fourth stage – “reincluding” – that is the focus of this paper.

## Results

### Reincluding

The intent of this truancy research was to learn how a sample of 13 youth experienced their world of returning to class after persistent truanting and the social aspects of their circumstances to understand how, and under what circumstances, NZ YwT are able to reintegrate in class after persistent truanting. To answer this question the findings from the fourth stage of the process theory of wagging, “reincluding” (Baskerville, 2019), are now presented.

“Reincluding” refers to the circumstances and support necessary for participants to make a positive change and to transition back to attending school regularly after truanting (Baskerville, 2019). This multidimensional construct contains two components – “accommodating” and “willing”’ – which indicate successful transitioning of students into regular school attendance and developing a sense of belonging in class. These components are now presented.

#### Accommodating

The term “accommodating” is used to explain the nature of teacher and peer interactions and the social structures that supported participants’ wagging cessation. Participants recognized that the support from teachers was different from peer support (Baskerville, 2021a). In their new schools, teachers provided meaningful contexts for learning, and dedicated more time to support participants’ learning needs:

I absolutely love [School B]. I never, ever could imagine a better school for me … I can’t pinpoint exactly what it is about School B but just everything, I love everything about this school.… I looked forward to class every single day and yeah, it was completely, completely different, and just so happy there, you know. I just absolutely, absolutely love it; I can’t imagine a better school for me. (Julie)

[Now I am] just succeeding in different things. Like achieving, achieving my work, finishing off my maths that I never even used to fucken’ look at, we just used to look at it and draw a big black power fist on them. Now I’m writing numbers. (TM)

Here I get like all the attention ‘cause there’s not that much kids, like being in a small environment is better for me and like yeah it’s better than the last school. (Decity)

They help you with your work, they make sure you get what you need and they also pay attention to you and it’s not that easy to wag here. (James)

Attendance at a new activity center or an inclusive secondary school with intimate classroom environments supported participants to connect with learning, experience improved attendance, and progress their learning. Their experiences changed as they received academic support; they felt safe, stayed in class, and were motivated to attend. Teachers’ behaviour defined and maintained classroom roles and circumstances necessary for inclusive classrooms. According to these participants when teachers noticed them, cared, and set clear boundaries, wagging was no longer necessary. These deliberate relational practices restored participants’ “mana” (personal power) and opened a space for YwT to be safe and present in class and matter to others. Transition back to class was possible because teachers behaved respectfully to students, participants began to experience success, and learning became positive and transformative. Teachers’ accommodation to the previously excluded participants’ needs facilitated reincluding: teachers played an important role as mentors by modelling positive interactions and establishing positive classroom climates, which provided motivation for students to be psychologically present and participate in learning.

Sometimes other adults within the school had a role to play in accommodating participants’ needs. In the present study, counsellors provided a positive experience and a safe place for participants to talk:

It helps to know there is someone there. A counsellor is kind to me; respectful and listens. People try to offer help and it’s up to them whether they take it, or whether they don’t. (Christina)

I started seeing a counsellor and it’s starting to help: I’m more open. (Tyrone)

It’s kind of like you know there is always going to be someone at the Counsellor’s Office when others might not have time for you. (Christina)

The participants understood that receiving assistance was their choice. Someone being there was reassuring. Participants needed to know that people were available and accessible to them. They recognised that teachers were not always available to help them with problems, consequently specialist help from counsellors was necessary to meet their emotional needs. One-on-one time with counsellors provided a positive experience and supported participants to see things differently. Participants recognised from meetings that they mattered and that their opinions were important when counsellors listened to them. Adults provided a way forward when YwT experienced collaborative approaches that were free from judgment and blame and when trust built between adults and YwT. Assistance, in the form of kindness and attentiveness, built trust, encouraged openness and reassurance, and provided participants the continued support necessary for change. Accommodating in this context provides an opportunity for teachers to think about changes they can make in relational practices, moving from a punitive to an inclusive ideology. It was also very clear that participants understood what was needed to stop them wagging:

Knowing more about what was going on in student’s lives would help and I think teachers and staff need to discuss giving a helping hand. (Christina)

I think people should have treated me better, I just think that people have no respect for one another, I think sometimes people forget. (Rose)

Maybe they need to try and find a friend in that class or try to fit in. (Christina)

People have no respect for one another. I think what would help is just having someone to stick up for them whether it’s a friend or whether it’s a teacher that won’t take it. Just someone that has your back is what everyone needs. (Rose)

In class, these girls were vulnerable and sensitive to others’ attitudes and recognized that classmates disrespected one another. They also recognized the importance of supportive friends in class. If friends were not in class, participants found alternative ways to fit in, which required a conscious effort. Participants understood that they needed acceptance, better treatment, and protection.

In summary, accommodating functioned to support participants to share, provided the circumstances to encourage participants to be the best they could be, and alleviated their vulnerability in the classroom so that they participated and developed future aspirations. When these aspects were fulfilled, participants experienced reincluding. The features of accommodating were: Dedication of teacher time to participants, strong trust-relationships between teachers and participants and between peers and participants, emotional support from counsellors, and safe inclusive classrooms with well-established routines. Under these conditions, participants were respected, felt secure, experienced social and academic support, and belonged as valued class members.

#### Willing

The term “willing” is used to describe student readiness, desire, and conviction to overcome previous challenges and the underlying factors that provided the impetus to make the essential changes to stop wagging (Baskerville, 2019). When participants had a reason to change and understood what they needed to do to make it happen, “willing” occurred:

The future ahead of me has changed my life [going to school] just seeing all those people succeeding and getting great careers and stuff. [I realised that] if I don’t change then I won’t get anywhere in the future. (James)

If you don’t get a good education you’re either going to wind up on the street, prison or no job. I don’t want to live on the street, live off a low wage or something. I want a good life. If I have a family in the future; I want a good life for them too. (Tyrone)

[It’s worth getting an education] so you can get a job and some money and a house, a car and a wife. (Michael)

Thinking about their future influenced participants to consider change. Noticing people succeeding, the importance of career opportunities, the desire for material possessions, and the reality of understanding the challenges of a life without education, influenced participants’ future aspirations. They recognized life without a good education is full of hardships and crime, and they wanted more out of life for themselves and future families. Witnessing others’ achievements and their desire for a good life, material possessions, and personal hope for the future were aspects of willing needed for change. Hope and/or a sense of agency was a necessary factor for participants to be willing to change so that they experienced reincluding:

They need to think that everything’s going to be ok, even if they don’t, they need to think that. (Christina)

Just myself realising that I needed to start changing. (James)

But I got there and I said to all my teachers, right so I want to be an art teacher, to do that I want to pass Level 1, 2 and 3 with merit endorsement at least, preferably with excellence and I want to get excellence in all my folios. (Julie)

When some participants knew what they wanted to do and understood how to get there, they developed a sense of agency and gained purpose. By imagining and positioning themselves in the future, some participants were able to envision future educational achievement, including the possibility of achieving excellence. For example, Julie told teachers she wanted to achieve. Willing required hope and imagination, positive thinking, future direction, scaffolding, accepting teacher support, and peer acceptance. The thought of achieving was motivating. However, finding motivation to change required participants to understand that it was in their best interests to take offered help. The participants who reincluded all had adult support.

To reposition themselves after truanting, participants in this study needed to experience purpose, a meaningful context for learning, self-belief, and peer approval. These conditions influenced them to develop the willingness to be open to change and experience reinclusion in class:

I should have been learning. Nah yeah I could have been learning ages ago, but yeah now I want to learn. (TM)

My Year 10 exams were like only a couple of weeks away so I stopped [wagging]. (Bob)

I don’t wag class anymore. I tell a teacher where I’m going. I listen to what I’ve been told by the school. (James)

Although it was up to participants to change their attitude towards wagging cessation, school life was difficult to manage without assistance from teachers. The participants wanted to change. When they were ready and eager to learn, understood the significance of missed opportunities, felt regret or made the decision to change, they co-operated with teachers and attended school again. By taking responsibility and acknowledging their part in wagging, students developed the desire to achieve and the willingness to make the required changes.

Participants also needed staff to listen for them to sustain the changes they were willing to make. Teacher support was crucial for successful reinclusion and for participants to stop wagging, communicate, and listen to teachers. Mutual respect, teacher advocacy, care and reciprocal listening were indicators of reincluding occurring in the classroom:

I had teachers actually say like I’m looking forward to seeing this when it’s finished and that they just really care and they get so involved and excited and just keep pushing you. I wanted to make them proud. (Julie)

Things are better. I get help now. She [the teacher] comes to me and checks out my work. She asks me what I need help with and shows me what to do. We work it out together. (Michael)

These participants saw teachers in their new schools as supportive and were impressed by the teachers’ attitudes and willingness to collaborate with them. Direct, articulate communication between teachers and participants contributed to participants’ ability to change. Having a vision and communicating that vision to teachers clearly contributed to participants’ willingness to change.

Participants understood what they were doing when they truanted but did not always know how to change (Baskerville, 2019). Adult guidance was required to change their positioning from failures to achievers. Willingness was evident when participants identified future aspirations, communicated their vision, and enlisted teacher support. This willingness was a crucial step in reinclusion. Willingness to change then served three functions for participants (a) to further develop hope, (b) to create the necessary desire and determination to overcome previous challenges, and (c) to find pathways forward that provided opportunities for them to envision success and attend class again.

## Discussion

This study delved deeper into the circumstances in NZ classrooms when YwT were reintegrated back to class after persistent truanting. During this reincluding stage, participants received social and academic support, care, and respect in their new schools, and were motivated to change, attend school again, and experience a successful outcome.

A range of supports for truanting students have been identified in the literature. For example, Frelin (2015) noted that deliberate relational practices ensure success for youth returning to class after truanting. That is, the teacher intentionally sets out to improve students’ negative self-image, repair the damage caused by past teachers by creating a different school atmosphere, and commit to improve the educational experiences of each student. Thus, Frelin’s research indicates that teacher intentionality is a core ingredient contributing to failing students’ social and academic success. Stahl and Dale (2013) found that inclusive learning environments involving positive peer relationships, equal status, peer approval, and meaningful contexts for learning provide a place for YwT to attend. Looking at a reintegration programme that offered a “clean slate,” Tootill and Spalding (2000) found that with support students could break away from negative feelings and experiences and respond to welcoming teachers and peers, and new curriculum learning opportunities. That is, the proper support contributed to their ability to change past behaviours and be reintegrated into learning (Tootill & Spalding, 2000).

Teachers’ perceptions of YwT matter. To improve their educational experience when returning to class post-truancy, YwT need an opportunity to get a “new start.” To reintegrate, YwT need teachers to be intentional in repairing harm from past dysfunctional relationships, in creating a positive classroom atmosphere, in respecting and caring for them, in providing academic support, and in showing them that they matter and their learning matters.

Peers also provide a way forward when YwT experience support and care. That is, when YwT connected with other YwT in a safe place of their choice outside class, they were positioned to be who they wanted to be, to relax, build trust, gain status, and be free of judgment and blame (Baskerville, 2021b). This peer bonding supported YwT to connect to others, alleviate insecurity and mistrust, matter to others, and belong. Outside class, in the presence of other YwT, previously unfulfilled classroom relationships were replaced by loyal friendships (Baskerville, 2021b). These positive peer interactions supported YwT to change their social identity so that, as a result, they experienced a superior social status outside the classroom compared to their previous inferior classroom status (Verhoeven & Poorthuis, 2019). According to Jones and Deutsch (2013), peer acceptance and friendship is important for academic performance, which is easily compromised when excluded (Juvonen et al., 2019). Similarly positive peer relationships influence youth ability to sustain attendance (Studsrød & Bru, 2011).

The research reported in this paper confirms that YwT need opportunities in classrooms to be accepted, not belittled; to be trusted, not suspected; to belong, not be disconnected. When teachers provide positive opportunities for peers to connect in classrooms, YwT may have opportunities to reconnect with teachers and learning in classrooms and improve their social identity. This is important in relation to reincluding YwT and their identity development – an important task for adolescences shaping the image of who they are and who they want to be (Verhoeven & Poorthuis, 2019).

In instances of reinclusion, teachers in this study indicated explicitly that YwT mattered, and attending class became the preferred activity. However, there is another essential factor in ensuring student sustain attendance after truanting: YwT need to be ready, willing and want to make this change. They need opportunities to look to the future, to aspire to do better, and to engage in meaningful contexts of learning relevant to their own lives. YwT perceive teachers’ lack of hope for them as meaning they are unworthy of their teachers’ attention (Bottrell, 2007). Conversely, teacher support to students to reengage with learning and to change their attitude towards attendance becomes an important aspect in students making an attitudinal change.

Clarity regarding career pathways and the qualifications required can provide students with a motivational platform for envisioning success and, therefore, engage teachers to support them and as a result to change their self-perception from a failure to a potential achiever (Freire et al., 2009). More broadly, changes in attitude can happen when the curriculum is meaningful and accessible, allowing youth to reposition themselves, moving from perceptions of under-achievement to experiencing success. To ensure the transition back to school is possible, YwT require an attitude of “willingness to change.”

Some participants reported that they were not receiving the support they needed to encourage them to return to the classroom. They understood that wagging meant that they missed learning and that this added to their struggle to understand work when they returned to class. However, the anticipation of being blamed for their absence when they returned to class was a large obstacle to overcome on their own. Teachers’ positive attitudes and support are important for welcoming students back to class and helping them build success and satisfaction in their learning, given (a) that a prevalent factor leading YwT to truancy is their perception that they have tenuous, uncaring relationships with their teachers and feel misunderstood (Bruce, 2018; Baskerville, 2021a); (b) that labelling YwT as a thing, which they do not agree with, oversimplifies their lived experience (Freire et al., 2009); and (c) that, according to Tarabini et al. (2018), teachers’ negative attitudes towards students greatly influence their diminished performance.

This study found that the support of a significant adult, not necessarily a teacher, could be important for helping students develop a positive and determined attitude to reengage with school. Mostly though, the findings confirm that both teachers and students play active roles in student reintegration in class after persistent truancy. These insights into effective approaches to managing and promoting school attendance in NZ schools may also provide approaches to addressing the educational inequity exasperated by decreased attendance in schools during the current global pandemic.

### Limits of the Study

The study is not without limitations. First, interview questions designed to ask participants about the cultural aspects of their lives did not emerge during the concurrent data collection and analysis process. The data contributing to this paper did not focus systematically on specific cultural aspects of the lives of participants who truanted classes. Furthermore, considering that cultural influences and students’ cultural background (class and ethnicity, in particular) are an important element of the similarities and differences that students experience and contribute to differential outcomes in education, including cultural considerations in further research would be beneficial. Specifically, to address this limitation, it is recommended time be spent clarifying the meaning of culture in relation to YwT, identify data relating to this, and gather more in-depth background information on each participant. Second, the sample size was small because YwT were hard to find, difficult to recruit, and not available for second interviews for various reasons. To address this limitation in further research, collaboration is recommended with schoolteachers to recruit additional students, which may also offer potential to gather both qualitative and quantitative data.

## Conclusion

The study investigated the process that occurred when participants truanted class in four New Zealand schools. As such, it extends truancy scholarship regarding the circumstances in New Zealand mainstream schools that supported participants in transitioning to attending school again, re-engaging in learning, and reintegrating in class after wagging. These insights add to Thomas’ (2015) claim that YwT no longer suffer exclusion when they experience positive feelings, success, and belonging. Further, the study provides evidence of why this shift in identity from failure to achiever occurs for YwT and the intentional teacher practices required for this to happen. That is, participants experienced reincluding when teachers dedicated time to them, facilitated the development of trusting relationships and established safe, inclusive learning environments, and when peers cared, supported and respected them. Under such conditions, participants experienced change – they developed hope, overcame previous challenges, envisioned success, and attended class again. It was through the accommodating role of teachers, peers, and the subsequent willingness of participants to make changes, that students engaged in reinclusion to school and experienced the shift in identity from failure to achiever.

Because this research involved particular youth in a very limited range of school settings, further studies are needed to replicate the design features in other contexts in order to confirm, modify, extend, or challenge the findings that emerged from this research. In addition, research using a different design, such as a mixed-methods quantitative and qualitative survey of different stakeholders (administrators, teachers, parents, and students), along with gathering and analysing observations of teachers’ practices, could be undertaken to examine particular provisions, practices, and values that are effective in preventing or reducing student wagging. A different research approach, building on the knowledge gained from the present study, is likely to add to our understanding of the phenomenon of wagging and how it may be prevented, or at least significantly reduced.
